# Do germanium-based photoinitiators have the potential to replace the well-established acylphosphine oxides?

**DOI:** 10.1039/d1dt02308j

**Published:** 2021-08-11

**Authors:** Tanja Wiesner, Michael Haas

**Affiliations:** Institute of Inorganic Chemistry, Graz University of Technology Stremayrgasse 9/IV 8010 Graz Austria michael.haas@tugraz.at

## Abstract

In the last few decades, there has been an increasing demand for photoinitiators with growing requirements. Nowadays, photoinitiators need to fulfill several requirements such as a low level of toxicity, biocompatibility, fast polymerization rates, high activities, good photobleaching and much more in order to remain competitive on the market. Accordingly, we compare acylphosphine oxides and acylgermanes, two common classes of photoinitiators, with respect to their various synthetic pathways, toxicity, availability and performance.

## Abstract 2

This frontier article highlights the importance of recently developed germanium-based photoinitiators in comparison with acylphosphine oxides. Their synthesis, performance, toxicity and availability are presented with advantages and drawbacks.

## Graphical abstract

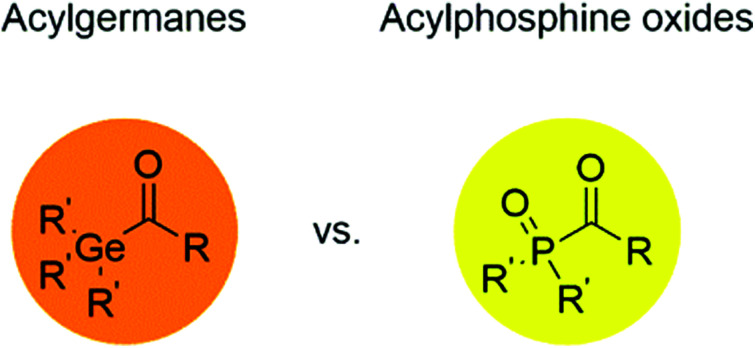

## Introduction

Photopolymerization has been an essential method for a variety of industrial processes for more than 30 years. Over the last century, there has been an immense increase in the demand and applications of photochemically produced high-performance polymers. Today, their use is no longer limited to the production of microelectronics, coatings, microlithography, optics and many more, but also applies in the medical field, *e.g.* as dental filling materials or for artificial tissues and for the production of 3D objects. As the demand for photoinitiators (PIs) has increased over the last few decades, the production of new initiators with improved properties is of not only great interest but also an enormous challenge.

The stringent requirements for various applications include fast polymerization rates, high activities, and excellent storage stability along with efficient photobleaching, sustainability, environmental compatibility and low to no toxicity of the PI and the photoproducts formed.^1,2^ Acylphosphine oxides represent a class of PIs that meet several of the abovementioned requirements. It is represented mainly by the monoacylphosphine oxides (MAPOs) and their bis(acyl) analogues (BAPOs). Due to their excellent properties, such as their high degree of whiteness, good thermal stability, and high curing efficiency, they almost seem unbeatable.^3^ However, they also come with some disadvantages. For instance, some acylphosphine oxide compounds have been described as toxic, which limits their applications in the medical field.^4,5^ In addition, some of them show limited solubility in aqueous media.^6^ However, Grützmacher and co-workers achieved a breakthrough in solubility with functionalized acylphosphine oxides.^7–10^

Recently, more and more germanium-based photoinitiators have been developed and investigated. Compared to phosphorus-based PIs, these initiators have a lower toxicity as well as a significant bathochromic shift of the longest wavelength absorption. However, not to be ignored is the low abundance of germanium in the Earth's crust, which leads to significantly higher costs of germanium-based PIs.^11–13^

Both compound classes, the acylphosphine oxides and germanium-based PIs follow a Norrish type-I cleavage mechanism upon light irradiation generating radicals and initiating the polymerization process. In the case of the former, a benzoyl- and a phosphinoyl-radical^14^ is generated, while in the case of the latter, a benzoyl- and a germyl-radical^15^ is generated. Type-I PIs can be defined as molecules that undergo homolytic bond cleavage in the triplet state and typically contain a benzoyl moiety as a chromophore. Primary radicals subsequently add to the double bond of an alkene initiating the polymerization. The quantum yield and the rate of addition of the initiator radicals to monomers are important for the efficiency of the reaction process.^2^

This frontier article focuses on the state-of-the-art synthetic methods of acylphosphine oxides and germanium-based PIs to examine their advantages and disadvantages. Toxicity as well as availability and performance as type-I PI for both compound classes will be discussed.

## Syntheses

### Acylphosphine oxides

**Scheme 1:**
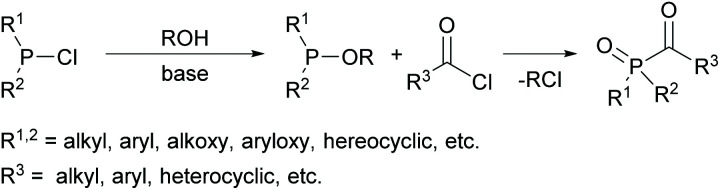
Classical synthetic route towards acylphosphine oxides.

Several commercially available MAPOs are readily obtained by an Arbuzov-type reaction of air- and moisture-sensitive alkoxyphosphanes with acyl chlorides (compare Scheme 1).^16,17^ However, the starting material for their synthesis are chlorophosphines, which are not easily available and their industrial production is a highly polluting process.^18^ Furthermore, the production of acylphosphine oxides gives rise to stoichiometric amounts of the toxic low-boiling alkyl chlorides, *e.g.* EtCl, as undesirable byproducts.^19^ These are released as volatile organic compounds (VOCs) that are difficult to handle and environmentally harmful.^20^

**Scheme 2:**
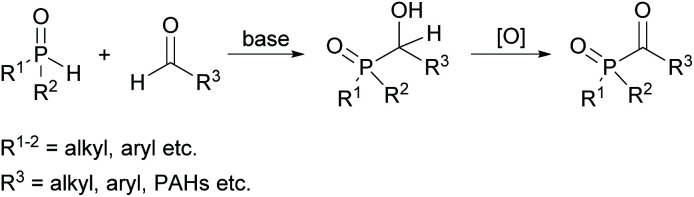
Synthetic route towards acylphosphine oxides through oxidation of α-hydroxyphosphine oxides.

An alternative route for the preparation of acylphosphine oxides is the addition of disubstituted phosphine oxides to aldehydes in the presence of bases and subsequent oxidation of the α-hydroxyphosphine oxides (shown in Scheme 2).^19,21^ The disadvantage of this process is the nevertheless often low oxidation efficiency, which leads to a high amount of oxidant consumption to achieve good yields.^22^

However, on the basis of this methodology, new acylphosphine oxides substituted with polycyclic aromatic hydrocarbons (PAHs) were recently investigated (Scheme 2).^23^ The driving force of this development is the availability of powerful light emitting diodes (LEDs), which emit light around 400 nm. Therefore, the introduction of these new acylphosphine oxides with bathochromic shifted absorption maxima is of high interest.

**Scheme 3:**
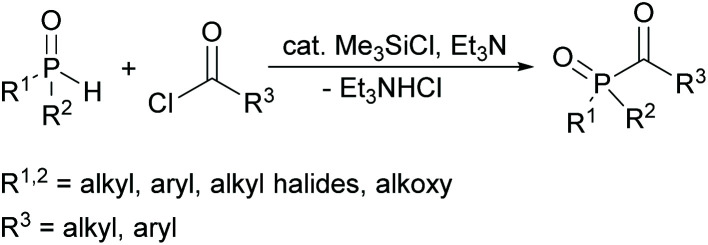
Synthetic route towards acylphosphine oxides using chlorosilanes as catalyst.

In this context, a new synthetic procedure for acylphosphine oxides was published by Han and Zhang nearly a year ago. Here a direct coupling of hydrogen phosphine oxides with acyl chlorides is mediated by chlorosilanes (see Scheme 3). The advantage of this synthetic route is the fact that it is environmentally friendlier and safer since no volatile alkyl halides are generated. It also eliminates the use of oxidizing agents used in conventional methods.^24^

**Scheme 4:**
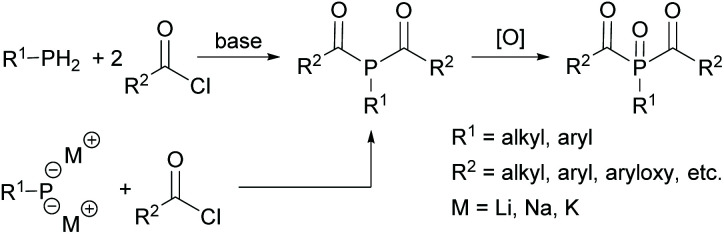
Synthetic route towards BAPOs starting from primary phosphanes or metallated derivatives.

While MAPOs are readily accessible through the reactions outlined above, the synthesis of BAPOs is more challenging. The typical synthetic routes to BAPOs involve the double acylation of either a primary phosphine (RPH_2_) or the corresponding metallated derivative (RPH_2−*x*_M_*x*_) with an acid chloride in the presence of a base. Subsequent oxidation with *e.g.* hydrogen peroxide leads to the corresponding bisacylphosphine oxide as shown in Scheme 4. However, this route suffers from inherent safety issues as well as low group functionality. In addition, only aryl or alkyl substituents can be attached to the phosphorus.^17,25^

**Scheme 5:**
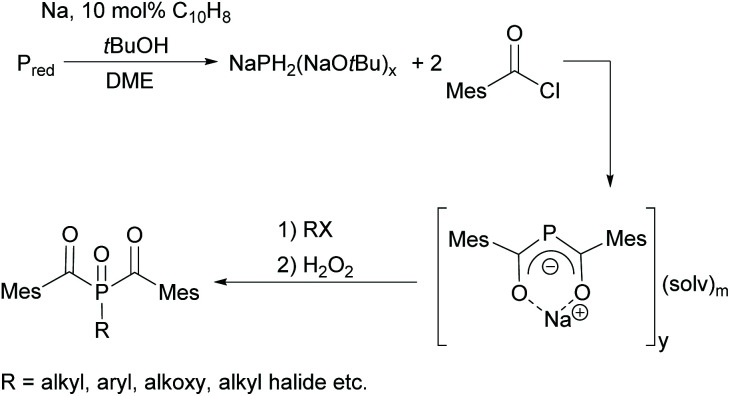
Synthetic route towards P-functionalized BAPOs.

Since acylphosphine oxides are mainly water-insoluble initiators, scientists are currently working on modifying the already known initiators. In recent years, the research group of Prof. Grützmacher has developed a convenient one-pot process for the conversion of elemental phosphorus to sodium bis(mesitoyl)phosphide NaP(COMes)_2_. For this purpose, a sodium *tert*-butylate aggregate NaPH_2_(NaOtBu)_*x*_ is first obtained from elemental phosphorus, sodium and *tert*-butanol.^26^ The subsequent reaction with mesitoyl chloride without prior isolation of the aggregate leads to the formation of NaP(COMes)_2_. A straightforward method for obtaining P-functionalized BAPO derivatives is provided by the nucleophilic substitution of NaP(COMes)_2_ with alkyl halides followed by oxidation (compare Scheme 5).^9,27^

**Scheme 6:**
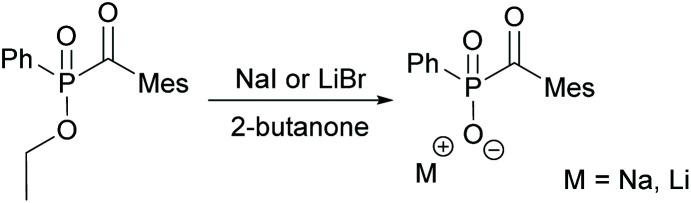
Synthesis of MAPO salts (Li–TPO and Na–TPO) starting from TPO–L.

With regard to the desired water solubility of acylphosphine oxides, both MAPO and BAPO salts represent new photoinitiators with these properties.^7,10,28^ The first research concerning this topic was performed by Schnabel *et al.* in 1991, where the water-soluble initiator lithium phenyl-2,4,6-trimethylbenzoyl phosphinate, which is a diphenyl(2,4,6-trimethylbenzoyl)phosphine oxide (TPO) derivative, was reported.^29^ The typical synthesis to MAPO salts starts from the commercially available 2,4,6-trimethylbenzoyl-ethoxylphenyl-phosphine oxide (TPO-L), which is synthesized by *e.g.* the procedure shown in Scheme 1. Further on, it is reacted with sodium iodide or lithium bromide, respectively, to give the corresponding MAPO salts, namely Li–TPO and Na–TPO (shown in Scheme 6).^29,30^

**Scheme 7:**
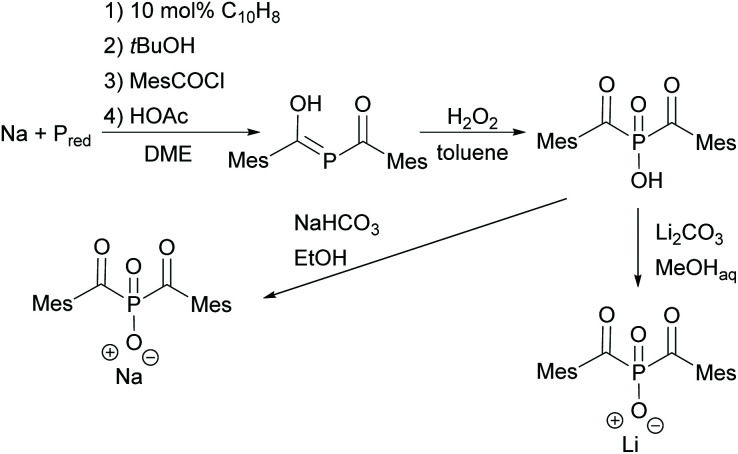
Synthesis of BAPO–OH and subsequent reaction to obtain BAPO salts (BAPO–ONa and BAPO–OLi).

In the case of BAPO, the bis(mesitoyl)phosphinic acid (BAPO–OH) is first prepared by the procedure already outlined above (compare Scheme 5). By the subsequent reaction with appropriate salts such as sodium hydrogen carbonate or lithium carbonate in low boiling alcohols, BAPO salts, namely BAPO–OLi^10^ or BAPO–ONa,^28^ can be prepared (shown in Scheme 7).

### Acylgermanes

**Scheme 8:**
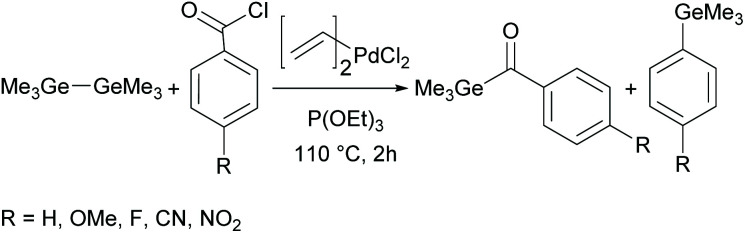
Synthesis of monoacylgermanes *via* palladium-catalysed cross-coupling.

Although acylgermanes have been known for more than 60 years, their excellent performance as free radical photoinitiators had long been overlooked. It was not until the seminal work of Liska and Moszner in 2008 with the implementation of acylgermanes as photoinitiators, that research in this field was revived.^31,32^ These monoacylgermanes were synthesized from hexamethyldigermane and acid chlorides in the presence of a Pd-catalyst and triethyl phosphite (see Scheme 8).

**Scheme 9:**
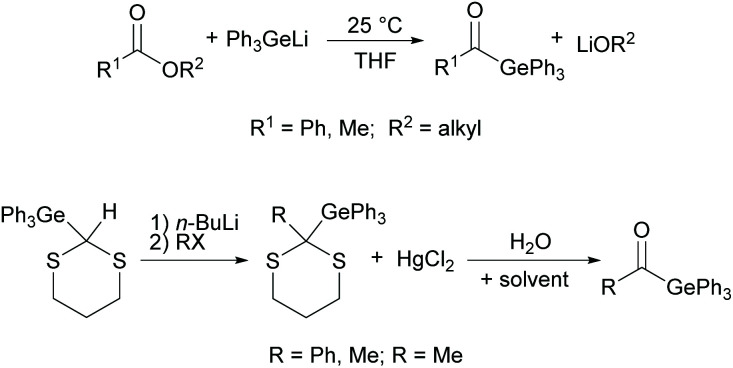
Synthesis of monoacylgermanes *via* germyl anions and Corey–Seebach approach.

A high activity was also reported for other monoacylgermanes.^33^ These compounds were synthesized by the reaction of Ph_3_GeLi with the respective ester,^34^ or from the corresponding germyl-1,3-dithiane (compare Scheme 9).^35^

**Scheme 10:**
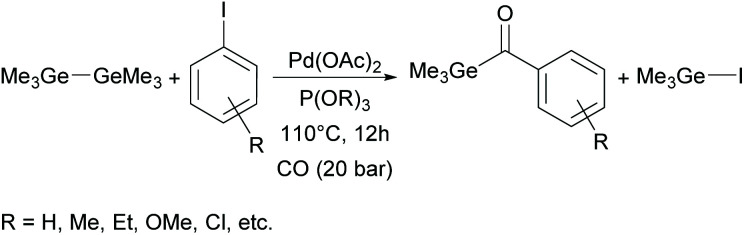
Synthesis of monoacylgermanes *via* palladium-catalysed carbonylative reaction.

Recently, Wu and co-workers implemented an alternative pathway towards monoacylgermanes.^36^ These monoacylgermanes were synthesized from hexamethyldigermane, carbon monoxide and (hetero)aryl iodides in the presence of a Pd-catalyst and triorgano phosphite (shown in Scheme 10).

**Scheme 11:**
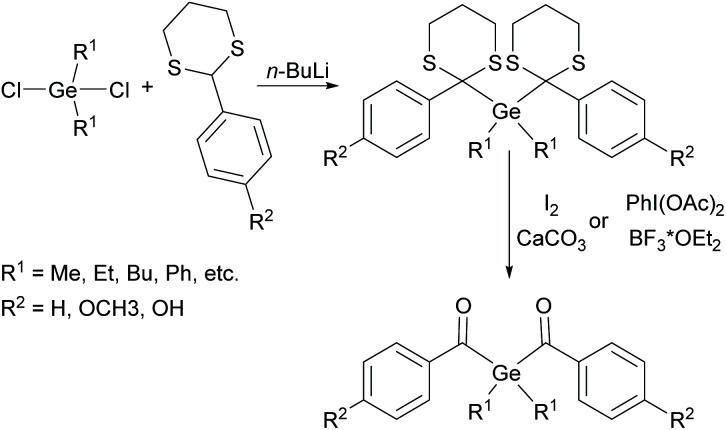
Synthesis of bisacylgermanes *via* Corey–Seebach approach.

Almost simultaneously with the introduction of monoacylgermanes as promising PIs, the investigation of bisacylgermanes was initiated. The synthetic protocol towards bisacylgermanes is based on a Corey–Seebach type reaction, which was adapted for higher homologues of carbon by A. Brook (compare Scheme 11).^32,35,37^

**Scheme 12:**
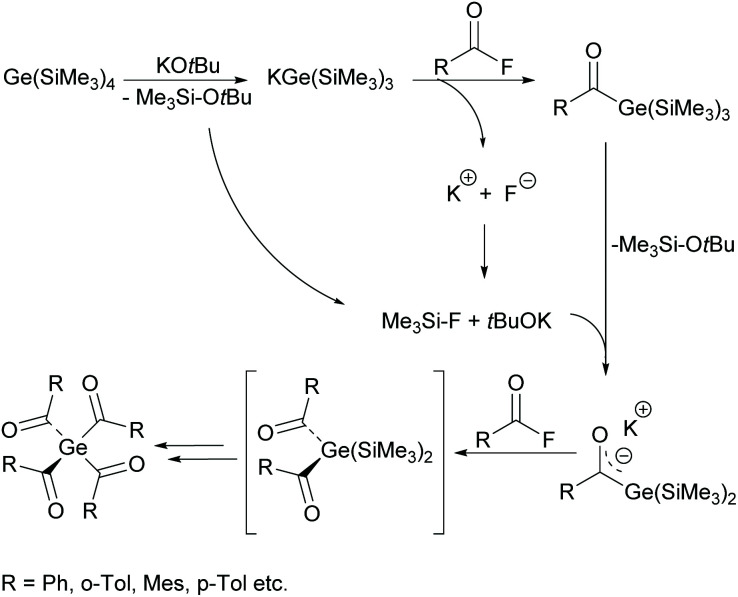
Synthesis of tetraacylgermanes *via* multiple silyl group abstraction methodology.

On the basis of the synthetic approach presented in Scheme 11, the first bisacylgermane was implemented as a commercially available photoinitiator [bis(4-methoxybenzoyl)diethylgermane= Ivocerin®]. In comparison with monoacylgermanes, bisacylgermanes show significantly enhanced extinction coefficients resulting in reduced curing times and increased curing depths of the final composite material.^11,38^ However, the multi-step synthesis and the tedious purification cause high production costs and prevent – so far – the application as PI apart from dental composites. In 2017 tris- and tetraacylgermanes were synthesized and implemented as long-wavelength PIs.^12,39^ During the course of these studies, a synthetic protocol allowing a straightforward access to these highly desirable compounds was developed (see Scheme 12). Here, the reaction of a tristrimethylsilyl-substituted germanide with 4 equivalents of acid fluorides leads to the formation of tetraacylgermanes *via* a multiple silyl abstraction methodology. Trisacylgermanes are formed *via* the same manner upon reaction of a bissilyl-substituted germanide with the respective acid fluoride.

**Scheme 13:**
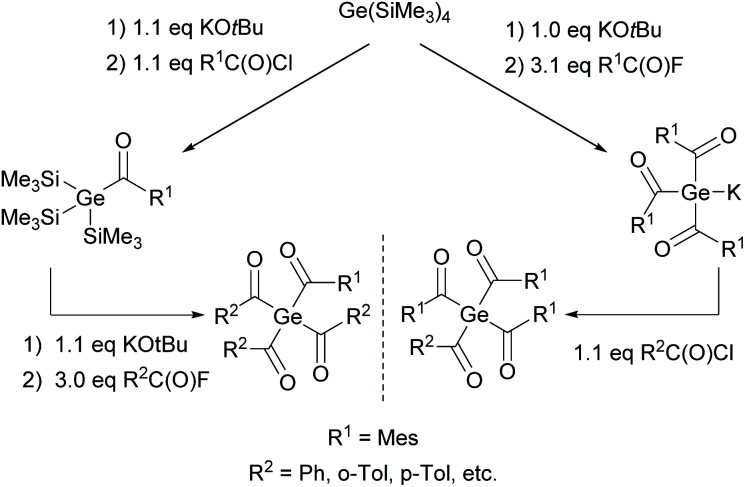
Synthesis of mixed-functionalized tetraacylgermanes.

Due to the presence of four RC=O chromophores, tetraacylgermanes show increased band intensities in comparison with bisacylgermanes, resulting in more efficient light absorption. A high group tolerance allows the tuning of the properties and shifting of the absorption band to higher wavelengths, which is ideal for medical applications. However, a major drawback of symmetrical tetraacylgermanes is their high melting points, which are responsible for low solubility, limiting the field of applications. Therefore, mixed-functionalized tetraacylgermanes have been implemented (compare Scheme 13).^12^ The introduction of different substituents on the germanium centre leads to increased solubility compared with symmetrical tetraacylgermanes.

## Toxicity

### Acylphosphine oxides

Testing of various commercially applied photoinitiators for their cytotoxicity as well as biocompatibility has become increasingly important in recent years, as the number of applications in the biomedical field has grown. Earlier this year, Xiao, Liu, Xing *et al.* published a report in which, among other photoinitiators, bis(2,4,6-trimethylbenzoyl)-phenylphosphine oxide (IRGACURE® 819), TPO and TPO-L were examined for their cytotoxicity, using MTT and CCK8 assays, and cytocompatibility towards four tissue types, in particular human cells, mouse cells, normal cells and cell lines. In this study, the tested PIs showed different degrees of cytotoxicity at concentrations ranging from 1 to 50 μM under non-irradiation conditions. BAPO exhibited the highest cytotoxicity (IC_50_ = 26.68 μM; concentration leading to a reduction of cell viability to 50%) among the seven PIs, while TPO appears cytotoxic in a concentration-dependent manner but with much lower toxicity than that of BAPO. TPO–L, together with another PI, showed the lowest cellular toxicity and at the same time better cytocompatibility with excellent transparency. Upon exposure to 455 nm blue light, these PIs resulted in increasing cytotoxicity to varying degrees, consistent with the trend towards non-irradiation conditions.^5^

In comparison, the water-soluble MAPO and BAPO salts, which were tested for cytotoxicity several years ago, were found to be virtually non-toxic. They were described by their LC_50_ (lethal concentration), *i.e.* the concentration required to kill half of the members of a tested population, in this case a cell culture, after a certain test duration, specifically 24 h. During this study, only Na–TPO was found to have very low biocompatibility (LC_50_ < 0.56 mM). In contrast, the cytotoxicity of Li–TPO (LC_50_ = 3.1 mM), BAPO–ONa (LC_50_ = 2.8 mM), and BAPO–OLi (LC_50_ = 2.6 mM) was found to be very low.^10^

According to ECHA (European Chemicals Agency), TPO is classified as Repr. (reproductive toxicity) 2 H361f, indicating that it is suspected of damaging fertility or the unborn child and causing atrophy of the testes. The classification is included in Annexure VI of the CLP (Classification, Labelling and Packaging) regulation.^40^ TPO is listed in the Community Rolling Action Plan (CoRAP), which means that the member-state Sweden will re-evaluate it in 2022, as it is proposed to further investigate reproductive toxicity and endocrine disrupting properties, persistence, terrestrial bioaccumulation and ecotoxicological properties.^41^ ECHA has indicated that they will increasingly use grouping of similar substances, as this is an alternative approach to fill data gaps in registrations submitted under REACH. This approach uses relevant information from analogous substances to predict the properties of target substances. This could of course mean that compounds such as BAPO (IRGACURE® 819) and TPO–L are grouped together with TPO in the dossier review, leading to subsequent restrictions in their use.^42^

### Acylgermanes

Ge-based photoinitiators are repeatedly described in the literature as only very slightly toxic to non-toxic.^11,13,32^ In 2008, for example, Ivocerin® was found to be non-cytotoxic. Also, the bacterial reverse mutation test (so-called Ames test) revealed that the compound did not induce gene mutations.^31,32,43^

In compliance with the data presented, Ivocerin® has also no harmonized classification according to ECHA and there are no reported hazards from manufacturers, importers or downstream users for this substance.^43^

## Availability

Phosphorus, the 12th most common element in the Earth's crust, is fundamental for life on Earth. It is crucial for the formation of DNA, cell membranes and bones. It is vital for food production since it is one of three nutrients (nitrogen, potassium, and phosphorus) used in commercial fertilizers. Consequently, high amounts of phosphorus are consumed every year for food production. Moreover, the Earth's population is growing each year and so is the demand for phosphorus. On the basis of these facts, there has been an ongoing debate about whether or not we are running out of phosphorus. However, this debate was largely dismissed after the United States Geological Survey (USGS) and other organizations increased world estimates on available phosphorus resources in 2021.^44^ Nevertheless, exact reserve quantities remain uncertain, as does the possible impacts of increased phosphate use on future generations. As outlined in numerous papers about germanium-based photoinitiators, the low abundancy of germanium in the Earth's crust (1.6 ppm) results in the high price of these new type of initators.^45^ Additionally, germanium is recovered mainly as a by-product from sphalerite zinc ores where it is concentrated in amounts up to 0.3%.^46^ Since germanium is not a primary resource, the market price of germanium metal is highly fluctuating. Therefore, these initiator systems cannot fully meet the requirements for photoinitiators in high-throughput polymer synthesis.

## Performance

**Scheme 14:**
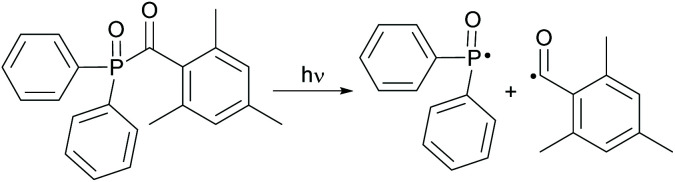
Photocleavage of type-I PIs on the example of TPO generating benzoyl- and phosphinoyl-radicals.

**Scheme 15:**
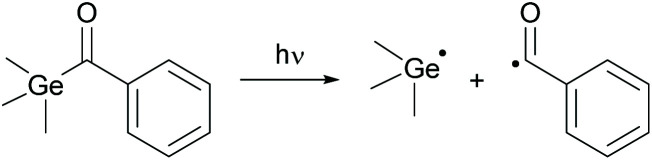
Photocleavage of type-I PIs on the example of benzoyltrimethylgermane generating benzoyl- and germyl-radicals.

Acylphosphine oxides as well as acylgermanes are so called Norrish type-I photoinitiators or one-component systems. Upon photocleavage, a benzoyl radical and, in the case of acylphosphine oxide compounds, a phosphinoyl-radical^14^ or, in the case of acylgermanes, a germyl-radical^15^ are generated. The radical formation is caused by an α-cleavage from the excited triplet state after photoexcitation and intersystem crossing (ISC) (compare Schemes 14 and 15). In both the cases, the phosphinoyl- as well as the germyl-radicals react multiple times faster than the benzoyl radical in the conversion of acrylate double bonds.^2,3^ Notably, germyl-radicals show significantly higher reactivity towards monomers than the related phosphinoyl-radicals.^27^

The symmetry forbidden n–π* transitions are responsible for the photoinduced α-cleavage of the Ge–C(O) or P–C(O) bond and thus the formation of reactive radical sites. In light-induced free radical polymerization, it is important that the emission spectrum of the LED- or UV-lamp overlaps with the absorption spectrum of the corresponding PI. Acylphosphine oxides show low absorption in UV-Vis spectroscopy, around 370–420 nm. Above ∼420 nm, respectively, no absorption is observed for these compounds, making these initiators unsuitable for long wavelength curing applications. The MAPO and BAPO salts, *i.e.* Na–TPO, Li–TPO, BAPO–ONa, BAPO–OLi, in turn have their absorption maxima of the n–π* transition approx. at 380 nm. Additionally, they show strong absorption bands well above 400 nm. When irradiated with visible light (400–500 nm), both MAPO and BAPO salts show high reactivity.^10^

In contrast to acylphosphine oxides, acylgermanes exhibit a significant red shift of the longest wavelength absorption. Benzoyltrimethylgermane, for example, exhibits a red shift of 30 nm compared to TPO, *i.e.*, from a *λ*_max_ value of 380 nm for the phosphorus compound to a *λ*_max_ value of 411 nm for the germanium compound.^37^ Accordingly, acylgermanes, such as tetraacylgermanes or dibenzoyldiethylgermanes, show long-wavelength absorption bands with *λ*_max_ values between 363 and 419 nm, which extend far into the visible range.^13^ This is particularly important for the photopolymerization of biocompatible materials, which require non-toxic PIs and non-toxic irradiation sources (visible light).

The high reactivity and excellent efficiency make the two classes of compounds well-suited photoinitiators for industrial purposes. Both initiator systems impress by excellent deep curing and high quantum yields of decomposition.

## Conclusions

To answer the title question “do germanium-based photoinitiators have the potential to replace the well-established acylphosphine oxides?”, yes and no, as both compound classes have their advantages and drawbacks. On the one hand, acylphosphine oxides exhibit cytotoxicity and will be examined more closely by ECHA in the future. On the other hand, they are cost-effective and have been established in industry for decades for high-throughput polymer synthesis. In comparison, acylgermanes have no or only very low cytotoxicity, but germanium is very expensive due to its low abundance in the Earth's crust. Consequently, acylgermanes have so far been only implemented as photoinitiators for medical applications. However, improved synthetic pathways and better availability may allow acylgermanes to compete with acylphosphine oxides. The future will show in which direction the development of photoinitiators will move, and it only requires to wait and research.

## Conflicts of interest

There are no conflicts to declare.

## Supplementary Material
